# Childhood infectious diseases and old age cognitive functioning: a nationally representative sample of community-dwelling older adults

**DOI:** 10.1017/S1041610220001404

**Published:** 2021-01

**Authors:** A. Rotstein, S. Z. Levine

**Affiliations:** Department of Community Mental Health, Faculty of Social Welfare and Health Sciences, University of Haifa, Haifa, Israel

**Keywords:** cognition, childhood illness, epidemiology

## Abstract

**Background:**

Cumulative evidence suggests that health-related risk factors during midlife and old-age are associated with cognitive impairment. However, studies are needed to clarify the association between early-life risk factors and impaired cognitive functioning to increment existing knowledge.

**Objective:**

To examine the association between childhood infectious diseases and late-life cognitive functioning in a nationally representative sample of older adults.

**Participants:**

Eligible respondents were 2994 community-dwelling individuals aged 65–85.

**Measurements:**

Cognitive functioning was assessed using the Mini-Mental State Examination (MMSE). Childhood infectious diseases (i.e. chicken pox, measles, and mumps) were self-reported. The study covariates were age, sex, highest educational level achieved, smoking status, body mass index, and depression. The primary statistical analysis examined the association between the number of childhood infectious diseases and total MMSE scores, accounting for all study covariates. Regression models of progressive complexity were examined for parsimony. The robustness of the primary results was tested in 17 sensitivity analyses.

**Results:**

The most parsimonious model was a linear adjusted model (Bayesian Information Criterion = 12646.09). Late-life cognitive functioning significantly improved as the number of childhood infectious diseases increased (*β* = 0.18; 95% CI = 0.11, 0.26; *p* < 0.001). This effect was not significantly attenuated in all sensitivity analyses.

**Conclusion:**

The current study results are consistent with prior ecological findings indicating that some childhood infectious diseases are associated with better cognitive functioning in old-age. This points to an early-life modifiable risk factor associated with older-life cognitive functioning. Our results may reflect selective mortality and/or beneficial effects via hormetic processes.

## Introduction

Impaired cognitive functioning among community-dwelling older adults is estimated to have a prevalence of 18.3% in the UK (Rait *et al.*, 2005), ranging between 8.7% and 22.2% in the United States (Plassman *et al.*, 2008; Langa *et al.*, 2008). Impaired cognitive functioning is associated with increased risks of Alzheimer’s disease (Bäckman *et al.*, 2005) and mortality (Langa *et al.*, 2008). The identification of risk factors and preventative strategies are primary objectives in research on impaired cognitive functioning in older adults (Brayne, 2007; Treichler and Jeste, 2019). Indeed, general health-related risk factors have been identified during midlife and old age (Launer, 2005; Launer, 2007; Kaufman and Perales-Puchalt, 2019; Lee *et al.*, 2019; Shaaban *et al.*, 2019; Tu *et al.*, 2020; Walker *et al.*, 2019). Nonetheless, a recent shift has seen studies that examine the role of early-life risk factors to increment current knowledge of risk factors (Grainger *et al.*, 2019; Launer, 2007; Whalley *et al.*, 2006; Williamson and Leroi, 2019).

Few observational studies have examined the association between health in early-life and late-life cognition. Two studies found that retrospective reports of better global childhood health were associated with improved cognitive functioning in old age (Luo and Waite, 2005; Yi *et al.*, 2007). Three others studies reported a null association (Barnes *et al.*, 2012; Yount, 2016; Zhang *et al.*, 2018). A single study examined the association between childhood infectious diseases and late-life cognitive functioning and showed mixed disease effects (Case and Paxson, 2009). This study used an ecological design to examine childhood health based on region-level historical data and linked it with performance on cognitive tests during adulthood. Ecological studies are valuable for assessing the global disease burden, yet may not accurately reflect the true association for a given group (Lilienfeld, 1983; Piantadosi *et al.*, 1988). In addition, ecological studies raise concerns of the ecological fallacy (Robinson, 1950), whereby lower and higher levels of abstraction are not interchangeable (i.e. implying individual-level characteristics from collective ones may be erroneous).

Competing views exist concerning the long-term impact of childhood infectious diseases. Exposure to childhood infectious diseases has been shown to be associated with both negative (Case and Paxson, 2009; Dalman *et al.*, 2008) and positive health outcomes in later life (Alexander *et al.*, 2000; Amirian *et al.*, 2016; Case and Paxson, 2009). Negative outcomes may be explained via direct effects of pathogens and indirect effects of inflammatory response (Khandaker *et al.*, 2012). Positive outcomes may be attributed to mild stress-induced repeated stimulation of protective mechanisms in cells and organisms which has a wide range of health-promoting and life span-extending effects (Rattan, 2008), and/or selective mortality.

The current study aims to examine the direction of the association between common childhood infectious diseases and late-life cognitive functioning in a nationally representative sample of community-dwelling older adults.

## Methods

### Design and data source

The current study cohort was based on a nationally representative sample of community-dwelling individuals aged 65 and over from the Republic of Ireland. Data were obtained from the Irish Longitudinal Study on Ageing (Kenny, 2018). This large-scale, nationally representative, aging study was conducted in Ireland. The current data were derived from interviews, undertaken between January 2016 and December 2016. The response rate was 62.0%. The study was approved by the Faculty of Health Sciences Research Ethics Committee in Trinity College Dublin. Further details of sampling and study design have been described elsewhere (Whelan and Savva, 2013).

### Participants

Eligible respondents for this study included community-dwelling individuals aged 65–85 (*N* = 3434). People with known or suspected dementia were not recruited. A subsample of people who did not have any information on past illnesses of any sort were removed (*N* = 440). Therefore, the final sample was 2994 people.

### Cognitive functioning

Cognitive functioning was assessed with the Mini-Mental State Examination (MMSE) (Folstein *et al.*, 1975). The MMSE is one of the most widely used research and screening measures of cognitive functioning in aging populations (Folstein *et al.*, 1975, 1983). It is easy to administer, available in over 15 languages (Steis and Schrauf, 2009), and has high levels of acceptability by health professionals and researchers as a diagnostic instrument (Nieuwenhuis-Mark, 2010). The scale has 20 items to assess orientation, recall, attention, calculation, language, and visuospatial abilities. It is divided into two sections, the first of which requires vocal responses only and covers orientation, memory, and attention; the maximum score is 21. The second part tests cognitive abilities to name, follow verbal and written commands, write a sentence spontaneously, and copy a polygon; the maximum score is nine. The maximum overall score is 30, with higher scores representing better cognitive functioning (Folstein *et al.*, 1983). Questions were asked and scored immediately. The test was not timed. The tester was instructed first to make the individual comfortable, to establish rapport, to praise successes, and to avoid pressing on items which the individual finds difficult.

In the current study, MMSE scores were computed, based on the original instructions (Folstein *et al.*, 1975) for each of the five subscales: orientation, registration, attention and calculation, recall, and language. Total scores were computed as well. For the primary analysis, the total test scores were not dichotomized into categories of impaired cognitive functioning and intact cognitive functioning because dichotomizing leads to information lost, increased risk of a false positive and may seriously underestimate the extent of variation in outcome such that individuals close to but on opposite sides of the cut point are characterized very differently rather than similarly (Altman and Royston, 2006). However, for robustness, when recomputing the sensitivity analysis, the total test scores were dichotomized into categories of impaired and intact cognitive functioning.

### Childhood infectious diseases

The childhood infectious diseases selected for this study were chickenpox, measles, and mumps. These were chosen because they are viral diseases, infection with which are followed by an enduring immunity (Simpson, 1952) and were common at the time of our sample’s childhood years due to recurrent outbreaks (London and Yorke, 1973). Childhood infectious diseases were assessed self-reported by asking participant if they have had chicken pox, measles, and mumps in childhood. Responses were classified as “yes” (score of 1), “no” (score of 0), or “don’t know” (coded as missing values). A total number of childhood infectious diseases score was computed for each person as the sum of the reported diseases of chicken pox, measles, and mumps. The total score ranged from 0 (having had no diseases) to 3 (having had all three diseases).

### Covariates

The following covariates were included in the analyses to adjust for potential confounding, model effect modification and to define subgroups of particular interest with possible differential effects on cognitive functioning. The covariates considered (and entered in the following order) were demographics, metabolic, and psychiatric. Demographic covariates were age at the time of data collection and sex (van der Flier and Scheltens, 2005), highest education achieved [primary or none, secondary, third, or higher (Solfrizzi *et al.*, 2004; Tervo *et al.*, 2004; Tyas *et al.*, 2007)], and smoking status at the time of data collection [classified as smoker or non-smoker (Swan and Lessov-Schlaggar, 2007)]. The metabolic covariate was body mass index categories [0–25, 25–30, 30–40, 40+ (Atti *et al.*, 2008)]. The psychiatric variable was depression (Butters *et al.*, 2008) as measured by the Composite International Diagnostic Interview (Robins *et al.*, 1988). This established instrument is widely used for assessing a clinical diagnosis of major depression in epidemiological and clinical studies. Respondents received binary scores depending on whether they had fulfilled criteria for a major depressive episode in the last 12 months or not.

### Analytic approach

First, missing values were examined, and the data were imputed accordingly using MICE (Multivariate Imputation by Chained Equations; van Buuren and Groothuis-Oudshoorn, 2010) in R package for multivariate imputation by chained equations. Second, sample characteristics were computed.

Third, the primary statistical analysis tested the association between the total MMSE scores as a function of the number of childhood infectious diseases using regression models. The assumptions of the regression models were tested. Visual inspection of residual figures was performed in order to reveal deviations from homoscedasticity or normality. An inspection for normality of error terms followed using a histogram and probability plots of the residuals. Independence of the error term was examined through a scatter plot of residuals by the predicted values to show that no discernible association existed. Next, multiple regression analysis models were computed. Regression models were computed in ascending complexity and tested without adjustment and adjusted for confounding of age at the time of data collection, sex, highest education achieved, smoking status, body mass index, and depression. Models were numbered as follows. The first model accounted for a linear effect of the number of childhood infectious diseases on MMSE scores (model 1 hereafter). The second model accounted for a linear effect of the number of childhood infectious diseases, age, sex, education level, smoking status, body mass index, and depression on MMSE scores (model 2 hereafter). The third model accounted for a quadratic effect of the number of childhood infectious diseases on MMSE scores (model 3 hereafter). The fourth model accounted for a quadratic effect of the number of childhood infectious diseases, age, sex, education level, smoking status, body mass index, and depression on MMSE scores (model 4 hereafter). The fifth model accounted for a cubic effect of the number of childhood infectious diseases on MMSE scores (model 5 hereafter). The sixth model accounted for a cubic effect of the number of childhood infectious diseases, age, sex, education level, smoking status, body mass index, and depression on MMSE scores (model 6 hereafter).

Fourth, the six models were compared for parsimony based on the Bayesian Information Criterion (BIC) for model selection (Schwarz, 1978), similar to prior research (Rotstein *et al.*, 2018). Lower BIC values represent more parsimonious models and so are a better fit to the data. The best fitting model was then chosen based on the lowest BIC score and plotted using the ggplot2 library (Wickham, 2011). All analyses were computed in R (R Core Team, 2018).

Fifth, the robustness of the primary results was tested in 17 sensitivity analyses. The most parsimonious regression model was recomputed in subgroups with differential effects on cognitive functioning. First, the most parsimonious model was recomputed without sex as a covariate for females then males, since females are at greater risk for impaired cognitive functioning (van der Flier and Scheltens, 2005). Second, the most parsimonious model was recomputed for persons aged 65–75 then aged 75–85, since cognitive functioning is related to age (van der Flier and Scheltens, 2005). Third, the most parsimonious model was computed for each of the three diseases (i.e. chicken pox, measles, and mumps) separately to show their individual effect on cognitive functioning. Fourth, the most parsimonious model was computed using a saturated model in which dummy variables for one, two, or three childhood infectious diseases were entered as covariates. Fifth, the most parsimonious model was computed for each of the MMSE subscales (i.e. orientation, registration, attention and calculation, recall, and language) separately. Sixth, the most parsimonious model was computed without education to account for effects of mediation. Seventh, possible methodological confounders were considered. Although dichotomizing may be problematic (Altman and Royston, 2006), for robustness and because dichotomous models have increased clinical lure, the most parsimonious model was recomputed dichotomized to account for intact cognitive functioning versus impaired cognitive functioning. Impaired cognitive functioning was defined using a cutoff score of 1.5 standard deviations below the mean score of the MMSE (Palmer *et al.*, 2002). Additionally, the most parsimonious model was recomputed for observed data with missing values and compared to the primary analysis based on imputed data (Sterne *et al.*, 2009).

## Results

### Data imputation and sample characteristics

The data were imputed using multiple imputations because simulation studies showed the technique to be robust even if the data are not missing at random (Sinharay *et al.*, 2001). The analytic sample consisted of 2994 older adults. The sample had a mean age of 73.50 (SD = 6.18) and 54.2% (*N* = 1,622) were female. The average MMSE score was 28.37 (SD = 2.14). Most of the participants had a history of measles (89.7%; *N* = 2,684), chicken pox (67.8%; *N* = 2,029), and/or mumps (62.3%; *N* = 1,866). See Table 1 for all sample characteristics.

**Table 1.** Sample characteristics

	M (SD)/*N* (%)
Total Mini-Mental State Examination score (0–30)	28.37 (2.14)
History of chicken pox (score of 1)	2029 (67.8%)
History of measles (score of 1)	2684 (89.7%)
History of mumps (score of 1)	1866 (62.3%)
Total number of childhood infectious diseases (0–3)	2.20 (0.95)
Age (65–85 years)	73.50 (6.18)
Sex (male)	1372 (45.8%)
Education	Primary/none: 906 (30.3%) Secondary: 1119 (37.4%) Third/higher: 969 (32.4%)
Smoking status (smoker)	265 (8.85%)
Body mass index	0–24.99: 1001 (33.4%) 25–29.99: 1334 (44.6%) 30–39.99: 628 (21.0%) 40+: 31 (1.0%)
Depression (a major depressive episode in the last 12 months)	99 (3.3%)

### Primary statistical analysis: cognitive functioning and the number of childhood infectious diseases

**Figure 1.**
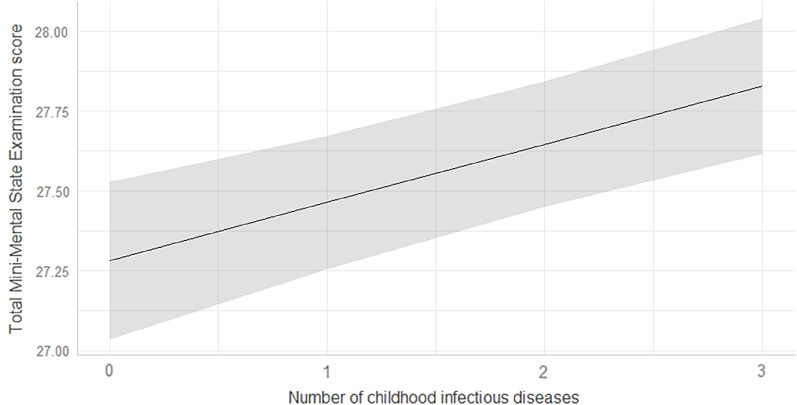
The linear adjusted effect of the number of childhood infectious diseases on Mini-Mental State Examination scores.

Note. For this adjusted model, having had one disease increases the Mini-Mental State Examination score by 0.18, all other covariates equal. Having had two diseases increases the Mini-Mental State Examination total score by 0.36, all other covariates equal. Having had three diseases increases the score by 0.54, all other covariates equal.

Comparison of the six regression models showed that model 2 was the most parsimonious (BIC = 12646.09; see supplementary Table S1 for comparison of BIC values for all regression models published as supplementary material online attached to the electronic version of this paper at https://doi.org/10.1017/S1041610220001404). This model accounted for the linear effect of the number of childhood infectious diseases on MMSE scores (Figure 1), adjusted for age, sex, education level, smoking status, body mass index, and depression. Late-life cognitive functioning improved as the number of childhood infectious diseases increased. Each disease incremented the MMSE score by 0.18. For two diseases, the MMSE total score increased by 0.36. Having had three diseases increased the MMSE score by 0.54. See Table 2 for model statistics.

**Table 2.**
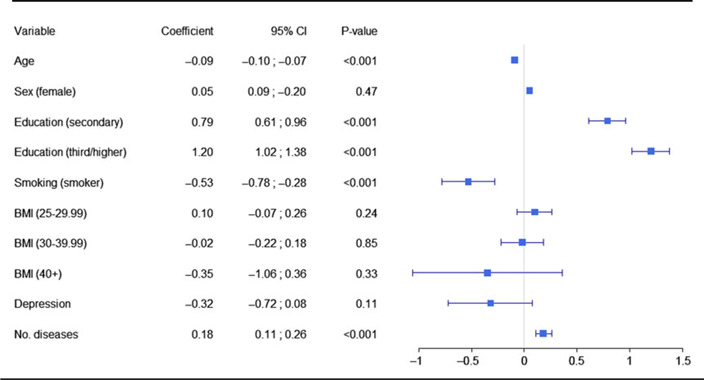
Model 2 statistics

Note. Model 2 is the linear effect of the number of childhood infectious diseases, age, sex, education level, smoking status, body mass index, and depression on Mini-Mental State Examination scores.

Sex reference group = male.

Education reference group = primary/none.

Smoking status reference group = non-smoker.

Body mass index reference group = 0–24.99.

Depression reference group = not having had a major depressive episode in the last 12 months.

Abbreviations: BMI = Body Mass Index; No. diseases = the number of childhood infectious diseases.

### Sensitivity analyses

Model 2, accounting for the linear effect of the number of childhood infectious diseases, age, education level, smoking status, body mass index, and depression on MMSE scores, was recomputed for all sensitivity analyses. A significant effect of childhood infectious diseases was found for males (*N* = 1372), females (*N* = 1622), persons aged 65–75 (*N* = 1828), persons aged 75–85 (*N* = 1166), each of the childhood infectious diseases separately (chicken pox, measles, and mumps), a saturated model (in which the effect size increased as the number of childhood infectious diseases increased), each subscale of the MMSE separately, a dichotomized regression model (intact cognitive functioning vs. impaired cognitive functioning), a model without education, and observed data with missing values (in which 191 people were excluded due to missingness; *N* = 2803). See supplementary Tables S2–S17 for model statistics (published as supplementary material online attached to the electronic version of this paper).

## Discussion

The current study examined the association between childhood infectious diseases and old age cognitive functioning, among community-dwelling older adults, using data from a representative national sample. The primary results show that late-life cognitive functioning improved as the number of childhood infectious diseases increased. Specifically, for each additional disease, there was an improvement in cognition reflecting a 0.18 MMSE total point increase. The primary result was not statistically significantly attenuated in a series of sensitivity analyses, including four subgroups with potentially differential cognitive functioning, two methodological confounders, two alternative models, each infectious disease and each MMSE subscale examined. The strongest effects were found among females and among those aged 75–85 years.

The current study results are consistent with prior ecological findings showing that some childhood infectious diseases (i.e. influenza) are associated with better cognitive functioning (i.e. successful counting) in old age (Case and Paxson, 2009). The literature provides related examples of early-life infectious diseases having a protective effect on health in later life. For instance, the varicella zoster virus that causes chickenpox is consistently reported to have an inverse association with glioma (Amirian *et al.*, 2016). Similarly, measles and/or combined childhood infections (chicken pox, measles, mumps, pertussis, and rubella) were found protective for Hodgkin’s disease (Alexander *et al.*, 2000).

Consistent with prior studies that have reported positive long-term health effects of childhood infectious diseases, our results contribute to identifying modifiable risk factors positively related to older-life cognitive functioning. Tentative explanations of our results are (I) selective mortality; and/or (II) that low levels of exposure to harmful agents may have beneficial effects via hormetic processes (Rattan, 2008). Future studies may identify possible mechanisms.

The current study has several limitations. First, selective mortality may have occurred, biasing the current study results. Second, our results are restricted to chickenpox, measles, and mumps. Future research is warranted to ascertain the effects of other childhood infectious diseases on cognitive functioning in old age. Third, childhood infectious diseases were assessed through self-reports which are less reliable than testing for serum antibodies (Mortimer, 1978). Specifically, higher rates of prevalence for actual mumps antibodies are expected since one person in three who contracts mumps does not present any symptoms (Mortimer, 1978). Still, prior evidence has suggested that these assessments of childhood health have reasonably good reliability and validity (Haas, 2007). In addition, past epidemiological studies have found similar prevalence rates as those reported in the current study for chickenpox, measles, and self-reported mumps (Mortimer, 1978; Pollock and Golding, 1993; Stocks, 1928). Nonetheless, it should be noted that a selection bias may have occurred as respondents with better cognitive function may have recalled their childhood experience of infectious diseases more accurately. Fourth, the MMSE relies on an interviewer administration and rating which may introduce further biases. Although the interviewer is given specific instructions for administration, differences resulting from the skill and style of the interviewer in eliciting answers and in scoring the answers given by the subject exist (Bowie *et al.*, 1999). Fifth, the sample did not include individuals with verified diagnoses of dementia or mild cognitive decline. Future studies may focus on individuals with and without dementia to extend the current findings.

## Conclusions

In summary, despite its limitations, the current study draws on a large representative sample and is the first to examine the individual and cumulative effects of common childhood infectious diseases (chickenpox, measles, and mumps) on late-life cognitive functioning. The study results show a consistent positive association between the number of childhood infectious diseases and late-life cognitive functioning. Childhood infectious diseases may be a future direction for modifiable risk factors related to older-life cognitive functioning.

## Supplementary material

For supplementary material accompanying this paper visit https://doi.org/10.1017/S1041610220001404. click here to view supplementary material

## References

[ref1] Alexander, F. E. et al. (2000). Risk factors for Hodgkin’s disease by Epstein-Barr virus (EBV) status: prior infection by EBV and other agents. British Journal of Cancer, 82, 1117–1121. 10737396 10.1054/bjoc.1999.1049 PMC2374437

[ref2] Altman, D. G. and Royston, P. (2006). The cost of dichotomising continuous variables. BMJ, 332(7549), 1080. 16675816 10.1136/bmj.332.7549.1080 PMC1458573

[ref3] Amirian, E. S. et al. (2016). History of chickenpox in glioma risk: a report from the glioma international case–control study (GICC). Cancer Medicine, 5, 1352–1358. 26972449 10.1002/cam4.682 PMC4924393

[ref4] Atti, A. R. et al. (2008). Late-life body mass index and dementia incidence: nine-year follow-up data from the Kungsholmen Project. Journal of the American Geriatrics Society, 56(1), 111–116. 18028342 10.1111/j.1532-5415.2007.01458.x

[ref5] Bäckman, L. et al. (2005). Cognitive impairment in preclinical Alzheimer’s disease: a meta-analysis. Neuropsychology, 19(4), 520. 16060827 10.1037/0894-4105.19.4.520

[ref6] Barnes, L. L. et al. (2012). Effects of early-life adversity on cognitive decline in older African Americans and whites. Neurology, 79(24), 2321–2327. 23233682 10.1212/WNL.0b013e318278b607 PMC3578376

[ref8] Bowie, P. et al. (1999). Should the Mini Mental State Examination be used to monitor dementia treatments? The Lancet, 354(9189), 1527–1528. 10.1016/S0140-6736(99)03486-8 10551506

[ref9] Brayne, C. (2007). The elephant in the room—healthy brains in later life, epidemiology and public health. Nature Reviews Neuroscience, 8(3), 233–239. 17299455 10.1038/nrn2091

[ref10] Butters, M. A. et al. (2008). Pathways linking late-life depression to persistent cognitive impairment and dementia. Dialogues in Clinical Neuroscience, 10(3), 345. 18979948 10.31887/DCNS.2008.10.3/mabutters PMC2872078

[ref11] Case, A. and Paxson, C. (2009). Early life health and cognitive function in old age. American Economic Review, 99(2), 104–109. 10.1257/aer.99.2.104 PMC4138129 25147383

[ref12] Dalman, C. et al. (2008). Infections in the CNS during childhood and the risk of subsequent psychotic illness: a cohort study of more than one million Swedish subjects. American Journal of Psychiatry, 165, 59–65. 10.1176/appi.ajp.2007.07050740 18056223

[ref13] Folstein, M. F. et al. (1975). “Mini-mental state”: a practical method for grading the cognitive state of patients for the clinician. Journal of Psychiatric Research, 12(3), 189–198. 1202204 10.1016/0022-3956(75)90026-6

[ref14] Folstein, M. F. et al. (1983). The mini-mental state examination. Archives of General Psychiatry, 40(7), 812. 6860082 10.1001/archpsyc.1983.01790060110016

[ref15] Grainger, S. A. et al. (2019). An investigation into early-life stress and cognitive function in older age. International Psychogeriatrics, 1–5. doi: 10.1017/S1041610219001583 31658915

[ref16] Haas, S. A. (2007). The long-term effects of poor childhood health: An assessment and application of retrospective reports. Demography, 44(1), 113–135. 17461339 10.1353/dem.2007.0003

[ref17] Kaufman, C. S. and Perales-Puchalt, J. (2019). Cardiovascular contributions to dementia: beyond individual risk factors. International Psychogeriatrics, 31(10), 1387–1389. 31657293 10.1017/S1041610219001285 PMC7158586

[ref18] Kenny, R. A. (2018). The Irish Longitudinal Study on Ageing (TILDA), 2012-2013.

[ref19] Khandaker, G. M. et al. (2012). Childhood infection and adult schizophrenia: a meta-analysis of population-based studies. Schizophrenia Research, 139(1–3), 161–168. 22704639 10.1016/j.schres.2012.05.023 PMC3485564

[ref20] Langa, K. M. et al. (2008). Trends in the prevalence and mortality of cognitive impairment in the United States: is there evidence of a compression of cognitive morbidity? Alzheimer’s and Dementia, 4(2), 134–144. 10.1016/j.jalz.2008.01.001 PMC2390845 18631957

[ref21] Launer, L. J. (2005). The epidemiologic study of dementia: a life-long quest? Neurobiology of Aging, 26(3), 335–340. 15639311 10.1016/j.neurobiolaging.2004.03.016

[ref22] Launer, L. J. (2007). Next steps in Alzheimer’s disease research: interaction between epidemiology and basic science. Current Alzheimer Research, 4(2), 141–143. 17430237 10.2174/156720507780362155

[ref23] Lee, E. E. et al. (2019). High prevalence and adverse health effects of loneliness in community-dwelling adults across the lifespan: role of wisdom as a protective factor. International Psychogeriatrics, 31(10), 1447–1462. 30560747 10.1017/S1041610218002120 PMC6581650

[ref25] Lilienfeld, A. M. (1983). Practical limitations of epidemiologic methods. Environmental Health Perspectives, 52, 3–8. 6653534 10.1289/ehp.83523 PMC1569326

[ref26] London, W. P. and Yorke, J. A. (1973). Recurrent outbreaks of measles, chickenpox and mumps: I. Seasonal variation in contact rates. American Journal of Epidemiology, 98(6), 453–468. 4767622 10.1093/oxfordjournals.aje.a121575

[ref27] Luo, Y. and Waite, L. J. (2005). The impact of childhood and adult SES on physical, mental, and cognitive well-being in later life. The Journals of Gerontology Series B: Psychological Sciences and Social Sciences, 60(2), S93–S101. 10.1093/geronb/60.2.s93 PMC2505177 15746030

[ref28] Mortimer, P. P. (1978). Mumps prophylaxis in the light of a new test for antibody. British Medical Journal, 2(6151), 1523–1524. 365288 10.1136/bmj.2.6151.1523 PMC1608816

[ref61] Nieuwenhuis-Mark, R. E. (2010). The death knoll for the MMSE: has it outlived its purpose? Journal of Geriatric Psychiatry and Neurology, 23(3), 151–157. 20231732 10.1177/0891988710363714

[ref29] Palmer, K. et al. (2002). Differential evolution of cognitive impairment in nondemented older persons: results from the Kungsholmen Project. American Journal of Psychiatry, 159(3), 436–442. 10.1176/appi.ajp.159.3.436 11870008

[ref30] Piantadosi, S. et al. (1988). The ecological fallacy. American Journal of Epidemiology, 127(5), 893–904. 3282433 10.1093/oxfordjournals.aje.a114892

[ref31] Plassman, B. L. et al. (2008). Prevalence of cognitive impairment without dementia in the United States. Annals of Internal Medicine, 148(6), 427–434. 18347351 10.7326/0003-4819-148-6-200803180-00005 PMC2670458

[ref32] Pollock, J. I. and Golding, J. (1993). Social epidemiology of chickenpox in two British national cohorts. Journal of Epidemiology and Community Health, 47(4), 274–281. 8228761 10.1136/jech.47.4.274 PMC1059792

[ref33] Rait, G. et al. (2005). Prevalence of cognitive impairment: results from the MRC trial of assessment and management of older people in the community. Age and Ageing, 34(3), 242–248. 15863409 10.1093/ageing/afi039

[ref34] Rattan, S. I. S. (2008). Hormesis in aging. Ageing Research Reviews, 7(1), 63–78. 17964227 10.1016/j.arr.2007.03.002

[ref35] Robins, L. N. et al. (1988). The Composite International Diagnostic Interview: an epidemiologic instrument suitable for use in conjunction with different diagnostic systems and in different cultures. Archives of General Psychiatry, 45(12), 1069–1077. 2848472 10.1001/archpsyc.1988.01800360017003

[ref36] Robinson, W. (1950). Ecological correlations and the behavior of individuals, American Sociological Review, 15, 351–357.

[ref37] Rotstein, A. et al. (2018). Age of onset and quality of life among males and females with schizophrenia: a national study. European Psychiatry, 53, 100–106. 29957368 10.1016/j.eurpsy.2018.06.004

[ref38] Schwarz, G. (1978). Estimating the dimension of a model. The Annals of Statistics, 6(2), 461–464.

[ref39] Shaaban, C. E. et al. (2019). Independent and joint effects of vascular and cardiometabolic risk factor pairs for risk of all-cause dementia: a prospective population-based study. International Psychogeriatrics, 31(10), 1421–1432. 31455442 10.1017/S1041610219001066 PMC6948010

[ref40] Simpson, R. H. (1952). Infectiousness of communicable diseases in the household (measles, chickenpox, and mumps). Lancet, 549–554. 12981903 10.1016/s0140-6736(52)91357-3

[ref41] Sinharay, S. et al. (2001). The use of multiple imputation for the analysis of missing data. Psychological Methods, 6(4), 317. 11778675

[ref42] Solfrizzi, V. et al. (2004). Vascular risk factors, incidence of MCI, and rates of progression to dementia. Neurology, 63(10), 1882–1891. 15557506 10.1212/01.wnl.0000144281.38555.e3

[ref62] Steis, M. R. and Schrauf, R. W. (2009). A review of translations and adaptations of the Mini-Mental State Examination in languages other than English and Spanish. Research in Gerontological Nursing, 2(3), 214–224. 20078011 10.3928/19404921-20090421-06

[ref43] Sterne, J. A. C. et al. (2009). Multiple imputation for missing data in epidemiological and clinical research: potential and pitfalls. BMJ, 338, b2393. 19564179 10.1136/bmj.b2393 PMC2714692

[ref44] Stocks, P. (1928). A study of the epidemiology of measles. Annals of Eugenics, 3(3–4), 361–398.

[ref45] Swan, G. E. and Lessov-Schlaggar, C. N. (2007). The effects of tobacco smoke and nicotine on cognition and the brain. Neuropsychology Review, 17(3), 259–273. 17690985 10.1007/s11065-007-9035-9

[ref46] R Core Team (2018). R: A Language and Environment for Statistical Computing, R Foundation for Statistical Computing. Austria: R Core Team.

[ref47] Tervo, S. et al. (2004). Incidence and risk factors for mild cognitive impairment: a population-based three-year follow-up study of cognitively healthy elderly subjects. Dementia and Geriatric Cognitive Disorders, 17(3), 196–203. 14739544 10.1159/000076356

[ref48] Treichler, E. B. and Jeste, D. V. (2019). Cognitive decline in older adults: applying multiple perspectives to develop novel prevention strategies. International Psychogeriatrics, 31(7), 913–916.

[ref49] Tu, L. et al. (2020). Trajectories of cognitive function and their determinants in older people: 12 years of follow-up in the Chinese Longitudinal Healthy Longevity Survey. International Psychogeriatrics, 32(6), 765–775. 32336299 10.1017/S1041610220000538

[ref50] Tyas, S. L. et al. (2007). Transitions to mild cognitive impairments, dementia, and death: findings from the Nun Study. American Journal of Epidemiology, 165(11), 1231–1238. 17431012 10.1093/aje/kwm085 PMC2516202

[ref51] van Buuren, S. and Groothuis-Oudshoorn, C. G. (2010). Mice: multivariate imputation by chained equations in R. Journal of Statistical Software, 1–68.

[ref52] van der Flier, W. M. and Scheltens, P. (2005). Epidemiology and risk factors of dementia. Journal of Neurology, Neurosurgery and Psychiatry, 76(Suppl. 5), v2–v7. 10.1136/jnnp.2005.082867 PMC1765715 16291918

[ref53] Walker, K. A. et al. (2019). Association of midlife to late-life blood pressure patterns with incident dementia. JAMA, 322(6), 535–545. 31408138 10.1001/jama.2019.10575 PMC6692677

[ref54] Whalley, L. J. et al. (2006). A life-course approach to the aetiology of late-onset dementias. The Lancet Neurology, 5(1), 87–96. 16361026 10.1016/S1474-4422(05)70286-6

[ref55] Whelan, B. J. and Savva, G. M. (2013). Design and methodology of the Irish Longitudinal Study on Ageing. Journal of the American Geriatrics Society, 61, S265–S268. 23662718 10.1111/jgs.12199

[ref56] Wickham, H. (2011). ggplot2. Wiley Interdisciplinary Reviews: Computational Statistics, 3(2), 180–185.

[ref57] Williamson, W. and Leroi, I. (2019). Thinking about dementia: is childhood too early? International Psychogeriatrics, 31(12), 1689–1690. 31856931 10.1017/S1041610219001157

[ref58] Yi, Z. et al. (2007). The association of childhood socioeconomic conditions with healthy longevity at the oldest-old ages in China. Demography, 44(3), 497–518. 17913008 10.1353/dem.2007.0033

[ref59] Yount, K. M. (2016). Gender, resources across the life course, and cognitive functioning in Egypt. In: N. Chamlou and M. Karshenas (Eds.), Women, Work and Welfare in the Middle East and North Africa (pp. 107–133). Singapore: World Scientific.

[ref60] Zhang, Z. et al. (2018). The long arm of childhood in China: early-life conditions and cognitive function among middle-aged and older adults. Journal of Aging and Health, 30(8), 1319–1344. 28645234 10.1177/0898264317715975

