# Supplementary material for: Childhood infectious diseases and old age cognitive functioning: a nationally representative sample of community-dwelling older adults
Source: Int Psychogeriatr. 2020 Jul 24;33(1):75–82. doi: 10.1017/S1041610220001404 (PMC8482372; doi:10.1017/S1041610220001404)
Supplement: Supplementary file 1 [file S1041610220001404sup001.docx]

**Childhood Infectious Diseases and Old Age Cognitive Functioning:**

**A Nationally Representative Sample of Community-Dwelling Older Adults**

**Supplemental material**

Contents

[Supplementary Table S1: Bayesian Information Criterion values of the six regression models 2](#_Toc41062303)

[Supplementary Table S2: Model 2 statistics for males 3](#_Toc41062304)

[Supplementary Table S3: Model 2 statistics for females 4](#_Toc41062305)

[Supplementary Table S4: Model 2 statistics for participants aged 65-75 5](#_Toc41062306)

[Supplementary Table S5: Model 2 statistics for participants aged 75-85 6](#_Toc41062307)

[Supplementary Table S6: Model 2 statistics for the individual effect of chicken pox on cognitive functioning 7](#_Toc41062308)

[Supplementary Table S7: Model 2 statistics for the individual effect of measles on cognitive functioning 8](#_Toc41062309)

[Supplementary Table S8: Model 2 statistics for the individual effect of mumps on cognitive functioning 9](#_Toc41062310)

[Supplementary Table S9: A saturated model with dummy variables for one, two, or three childhood infectious diseases 10](#_Toc41062311)

[Supplementary Table S10: Model 2 statistics for the Mini Mental State Examination orientation sub-scale. 11](#_Toc41062312)

[Supplementary Table S11: Model 2 statistics for the Mini Mental State Examination registration sub-scale. 12](#_Toc41062313)

[Supplementary Table S12: Model 2 statistics for the Mini Mental State Examination attention and calculation sub-scale. 13](#_Toc41062314)

[Supplementary Table S13: Model 2 statistics for the Mini Mental State Examination recall sub-scale. 14](#_Toc41062315)

[Supplementary Table S14: Model 2 statistics for the Mini Mental State Examination language sub-scale. 15](#_Toc41062316)

[Supplementary Table S15: Model 2 statistics for the dichotomized model (intact cognitive functioning vs. impaired cognitive functioning) 16](#_Toc41062317)

[Supplementary Table S16: Model 2 statistics without education 17](#_Toc41062318)

[Supplementary Table S17: Model 2 statistics for observed data with missing values 18](#_Toc41062319)

## Supplementary Table S1: Bayesian Information Criterion values of the six regression models

| **Model** | **Bayesian Information Criterion value** |
| --- | --- |
| Model 1 | 13018.01 |
| Model 2 | 12646.09* |
| Model 3 | 13023.58 |
| Model 4 | 12650.78 |
| Model 5 | 13030.93 |
| Model 6 | 12658.42 |

* most parsimonious model

Note.

Model 1 = The linear effect of the Mini Mental State Examination scores on the number of childhood infectious diseases.

Model 2 = The linear effect of the Mini Mental State Examination scores as a function of the number of childhood infectious diseases, age, sex, education level, smoking status, Body Mass Index and depression.

Model 3 = The quadratic effect of the Mini Mental State Examination scores on the number of childhood infectious diseases.

Model 4 = The quadratic effect of the Mini Mental State Examination scores as a function of the number of childhood infectious diseases, age, sex, education level, smoking status, Body Mass Index and depression.

Model 5 = The cubic effect of the Mini Mental State Examination scores on the number of childhood infectious diseases.

Model 6 = The cubic effect of the Mini Mental State Examination scores as a function of the number of childhood infectious diseases, age, sex, education level, smoking status, Body Mass Index and depression.

## Supplementary Table S2: Model 2 statistics for males

| Variable | β | [95% confidence interval] | | P value |
| --- | --- | --- | --- | --- |
| Age | -0.08 | -0.10 | -0.06 | <0.001 |
| Education (secondary) | 0.78 | 0.52 | 1.04 | <0.001 |
| Education (third/higher) | 1.24 | 0.98 | 1.51 | <0.001 |
| Smoking status (smoker) | -0.54 | -0.92 | -0.16 | 0.006 |
| Body Mass Index (25-29.99) | 0.13 | -0.12 | 0.39 | 0.33 |
| Body Mass Index (30-39.99) | -0.13 | -0.44 | 0.17 | 0.40 |
| Body Mass Index (40+) | -0.74 | -2.00 | 0.51 | 0.24 |
| Depression (a major depressive  episode in the last 12 months) | -0.56 | -1.32 | 0.20 | 0.15 |
| Total number of childhood infectious diseases | 0.158 | 0.04 | 0.26 | 0.004 |

Note.

N=1372

Model 2 = the linear effect of the number of childhood infectious diseases, age, education level, smoking status, Body Mass Index and depression on Mini Mental State Examination scores.

Education reference group = primary/none

Smoking status reference group = non-smoker

Body Mass Index reference group = 0-24.99

Depression reference group = not having had a major depressive episode in the last 12 months

## Supplementary Table S3: Model 2 statistics for females

| Variable | β | [95% confidence interval] | | P value |
| --- | --- | --- | --- | --- |
| Age | -0.10 | -0.11 | -0.08 | <0.001 |
| Education (secondary) | 0.79 | 0.55 | 1.03 | <0.001 |
| Education (third/higher) | 1.153 | 0.89 | 1.40 | <0.001 |
| Smoking status (smoker) | -0.53 | -0.86 | -0.19 | 0.002 |
| Body Mass Index (25-29.99) | 0.06 | -0.15 | 0.28 | 0.58 |
| Body Mass Index (30-39.99) | 0.09 | -0.17 | 0.36 | 0.50 |
| Body Mass Index (40+) | -0.16 | -1.02 | 0.69 | 0.71 |
| Depression (a major depressive  episode in the last 12 months) | -0.25 | -0.72 | 0.22 | 0.29 |
| Total number of childhood infectious diseases | 0.21 | 0.10 | 0.33 | <0.001 |

Note.

N=1622

Model 2 = the linear effect of the number of childhood infectious diseases, age, education level, smoking status, Body Mass Index and depression on Mini Mental State Examination scores.

Education reference group = primary/none

Smoking status reference group = non-smoker

Body Mass Index reference group = 0-24.99

Depression reference group = not having had a major depressive episode in the last 12 months

## Supplementary Table S4: Model 2 statistics for participants aged 65-75

| Variable | β | [95% confidence interval] | | P value |
| --- | --- | --- | --- | --- |
| Age | -0.05 | -0.08 | -0.03 | <0.001 |
| Sex (female) | 0.16 | 0.01 | 0.32 | 0.04 |
| Education (secondary) | 0.78 | 0.58 | 0.97 | <0.001 |
| Education (third/higher) | 1.10 | 0.90 | 1.30 | <0.001 |
| Smoking status (smoker) | -0.54 | -0.79 | -0.28 | <0.001 |
| Body Mass Index (25-29.99) | 0.14 | -0.04 | 0.32 | 0.12 |
| Body Mass Index (30-39.99) | -0.01 | -0.22 | 0.21 | 0.96 |
| Body Mass Index (40+) | -0.49 | -1.15 | 0.18 | 0.15 |
| Depression (a major depressive  episode in the last 12 months) | -0.15 | -0.55 | 0.24 | 0.44 |
| Total number of childhood infectious diseases | 0.08 | 0.00 | 0.17 | 0.05 |

Note.

N=1828

Model 2= the linear effect of the number of childhood infectious diseases, age, sex, education level, smoking status, Body Mass Index and depression on Mini Mental State Examination scores.

Sex reference group = male

Education reference group = primary/none

Smoking status reference group = non-smoker

Body Mass Index reference group = 0-24.99

Depression reference group = not having had a major depressive episode in the last 12 months

## Supplementary Table S5: Model 2 statistics for participants aged 75-85

| Variable | β | [95% confidence interval] | | P value |
| --- | --- | --- | --- | --- |
| Age | -0.14 | -0.17 | -0.10 | <0.001 |
| Sex (female) | -0.08 | -0.36 | 0.21 | 0.59 |
| Education (secondary) | 0.82 | 0.50 | 1.15 | <0.001 |
| Education (third/higher) | 1.36 | 1.01 | 1.70 | <0.001 |
| Smoking status (smoker) | -0.51 | -1.08 | 0.06 | 0.08 |
| Body Mass Index (25-29.99) | 0.03 | -0.28 | 0.34 | 0.85 |
| Body Mass Index (30-39.99) | -0.05 | -0.44 | 0.33 | 0.80 |
| Body Mass Index (40+) | 0.09 | -1.82 | 2.01 | 0.92 |
| Depression (a major depressive  episode in the last 12 months) | -0.83 | -1.72 | 0.07 | 0.07 |
| Total number of childhood infectious diseases | 0.32 | 0.18 | 0.47 | <0.001 |

Note.

N=1166

Model 2= the linear effect of the number of childhood infectious diseases, age, sex, education level, smoking status, Body Mass Index and depression on Mini Mental State Examination scores.

Sex reference group = male

Education reference group = primary/none

Smoking status reference group = non-smoker

Body Mass Index reference group = 0-24.99

Depression reference group = not having had a major depressive episode in the last 12 months

## Supplementary Table S6: Model 2 statistics for the individual effect of chicken pox on cognitive functioning

| Variable | β | [95% confidence interval] | | P value |
| --- | --- | --- | --- | --- |
| Age | -0.09 | -0.10 | -0.08 | <0.001 |
| Sex (female) | 0.06 | -0.08 | 0.21 | 0.39 |
| Education (secondary) | 0.80 | 0.62 | 0.97 | <0.001 |
| Education (third/higher) | 1.22 | 1.04 | 1.41 | <0.001 |
| Smoking status (smoker) | -0.54 | -0.80 | -0.29 | <0.001 |
| Body Mass Index (25-29.99) | 0.10 | -0.06 | 0.27 | 0.23 |
| Body Mass Index (30-39.99) | -0.02 | -0.22 | 0.18 | 0.88 |
| Body Mass Index (40+) | -0.36 | -1.07 | 0.35 | 0.32 |
| Depression (a major depressive  episode in the last 12 months) | -0.32 | -0.72 | 0.08 | 0.12 |
| Chicken pox | 0.26 | 0.10 | 0.41 | 0.001 |

Note.

N=2994

Model 2= the linear effect of chicken pox, age, sex, education level, smoking status, Body Mass Index and depression on Mini Mental State Examination scores.

Sex reference group = male

Education reference group = primary/none

Smoking status reference group = non-smoker

Body Mass Index reference group = 0-24.99

Depression reference group = not having had a major depressive episode in the last 12 months

## Supplementary Table S7: Model 2 statistics for the individual effect of measles on cognitive functioning

| Variable | β | [95% confidence interval] | | P value |
| --- | --- | --- | --- | --- |
| Age | -0.09 | -0.10 | -0.08 | <0.001 |
| Sex (female) | 0.06 | -0.09 | 0.21 | 0.42 |
| Education (secondary) | 0.78 | 0.61 | 0.96 | <0.001 |
| Education (third/higher) | 1.21 | 1.03 | 1.40 | <0.001 |
| Smoking status (smoker) | -0.54 | -0.79 | -0.29 | <0.001 |
| Body Mass Index (25-29.99) | 0.09 | -0.07 | 0.26 | 0.26 |
| Body Mass Index (30-39.99) | -0.03 | -0.23 | 0.17 | 0.76 |
| Body Mass Index (40+) | -0.41 | -1.11 | 0.30 | 0.26 |
| Depression (a major depressive  episode in the last 12 months) | -0.34 | -0.74 | 0.06 | 0.10 |
| Measles | 0.52 | 0.29 | 0.76 | <0.001 |

Note.

N=2994

Model 2= the linear effect of measles, age, sex, education level, smoking status, Body Mass Index and depression on Mini Mental State Examination scores.

Sex reference group = male

Education reference group = primary/none

Smoking status reference group = non-smoker

Body Mass Index reference group = 0-24.99

Depression reference group = not having had a major depressive episode in the last 12 months

## Supplementary Table S8: Model 2 statistics for the individual effect of mumps on cognitive functioning

| Variable | β | [95% confidence interval] | | P value |
| --- | --- | --- | --- | --- |
| Age | -0.09 | -0.10 | -0.08 | <0.001 |
| Sex (female) | 0.09 | -0.05 | 0.24 | 0.21 |
| Education (secondary) | 0.80 | 0.32 | 0.98 | <0.001 |
| Education (third/higher) | 1.22 | 1.04 | 1.41 | <0.001 |
| Smoking status (smoker) | -0.55 | -0.80 | -0.30 | <0.001 |
| Body Mass Index (25-29.99) | 0.10 | -0.07 | 0.26 | 0.24 |
| Body Mass Index (30-39.99) | -0.02 | -0.22 | 0.18 | 0.87 |
| Body Mass Index (40+) | -0.34 | -1.04 | 0.37 | 0.35 |
| Depression (a major depressive  episode in the last 12 months) | -0.33 | -0.73 | 0.07 | .011 |
| Mumps | 0.24 | 0.09 | 0.38 | 0.001 |

Note.

N=2994

Model 2= the linear effect of mumps, age, sex, education level, smoking status, Body Mass Index and depression on Mini Mental State Examination scores.

Sex reference group = male

Education reference group = primary/none

Smoking status reference group = non-smoker

Body Mass Index reference group = 0-24.99

Depression reference group = not having had a major depressive episode in the last 12 months

## Supplementary Table S9: A saturated model with dummy variables for one, two, or three childhood infectious diseases

| Variable | β | [95% confidence interval] | | P value |
| --- | --- | --- | --- | --- |
| Age | -0.09 | -0.10 | -0.08 | <0.001 |
| Sex (female) | 0.05 | -0.10 | 0.19 | 0.53 |
| Education (secondary) | 0.78 | 0.61 | 0.96 | <0.001 |
| Education (third/higher) | 1.20 | 1.01 | 1.38 | <0.001 |
| Smoking status (smoker) | -0.53 | -0.78 | -0.28 | <0.001 |
| Body Mass Index (25-29.99) | 0.09 | -0.07 | 0.26 | 0.26 |
| Body Mass Index (30-39.99) | -0.03 | -0.23 | 0.18 | 0.81 |
| Body Mass Index (40+) | -0.36 | -1.06 | 0.35 | 0.32 |
| Depression (a major depressive  episode in the last 12 months) | -0.32 | -0.72 | 0.08 | 0.12 |
| One childhood infectious disease | 0.31 | -0.01 | 0.63 | 0.058 |
| Two childhood infectious diseases | 0.59 | 0.29 | 0.90 | <0.001 |
| Three childhood infectious diseases | 0.64 | 0.35 | 0.93 | <0.001 |

Note.

N=2994

The saturated model is the linear effect of one, two, or three childhood infectious diseases, age, sex, education level, smoking status, Body Mass Index and depression on Mini Mental State Examination scores.

Sex reference group = male

Education reference group = primary/none

Smoking status reference group = non-smoker

Body Mass Index reference group = 0-24.99

Depression reference group = not having had a major depressive episode in the last 12 months

## Supplementary Table S10: Model 2 statistics for the Mini Mental State Examination orientation sub-scale.

| Variable | β | [95% confidence interval] | | P value |
| --- | --- | --- | --- | --- |
| Age | -0.01 | -0.02 | -0.01 | <0.001 |
| Sex (female) | 0.01 | -0.03 | 0.05 | 0.78 |
| Education (secondary) | 0.07 | 0.03 | 0.12 | 0.002 |
| Education (third/higher) | 0.11 | 0.06 | 0.16 | <0.001 |
| Smoking status (smoker) | -0.09 | -0.15 | -0.02 | 0.01 |
| Body Mass Index (25-29.99) | 0.03 | -0.02 | 0.07 | 0.24 |
| Body Mass Index (30-39.99) | -0.02 | -0.07 | 0.04 | 0.56 |
| Body Mass Index (40+) | -0.12 | -0.31 | 0.08 | 0.24 |
| Depression (a major depressive  episode in the last 12 months) | -0.13 | -0.24 | -0.03 | 0.01 |
| Total number of childhood infectious diseases | 0.02 | 0.0003 | 0.04 | 0.05 |

Note.

N=2994

Model 2= the linear effect of total number of childhood infectious diseases, age, sex, education level, smoking status, Body Mass Index and depression on scores of the Mini Mental State Examination orientation sub-scale.

Sex reference group = male

Education reference group = primary/none

Smoking status reference group = non-smoker

Body Mass Index reference group = 0-24.99

Depression reference group = not having had a major depressive episode in the last 12 months

## Supplementary Table S11**: Model 2 statistics for the Mini Mental State Examination registration sub-scale**.

| Variable | β | [95% confidence interval] | | P value |
| --- | --- | --- | --- | --- |
| Age | -0.001 | -0.002 | -0.0003 | 0.01 |
| Sex (female) | 0.01 | -0.004 | 0.02 | 0.16 |
| Education (secondary) | 0.02 | 0.003 | 0.03 | 0.02 |
| Education (third/higher) | 0.02 | 0.002 | 0.04 | 0.03 |
| Smoking status (smoker) | 0.003 | -0.02 | 0.03 | 0.80 |
| Body Mass Index (25-29.99) | 0.004 | -0.01 | 0.02 | 0.64 |
| Body Mass Index (30-39.99) | 0.01 | -0.01 | 0.03 | 0.30 |
| Body Mass Index (40+) | 0.02 | -0.05 | 0.08 | 0.55 |
| Depression (a major depressive  episode in the last 12 months) | 0.02 | -0.02 | 0.05 | 0.33 |
| Total number of childhood infectious diseases | 0.01 | 0.001 | 0.01 | 0.03 |

Note.

N=2994

Model 2= the linear effect of total number of childhood infectious diseases, age, sex, education level, smoking status, Body Mass Index and depression on scores of the Mini Mental State Examination registration sub-scale.

Sex reference group = male

Education reference group = primary/none

Smoking status reference group = non-smoker

Body Mass Index reference group = 0-24.99

Depression reference group = not having had a major depressive episode in the last 12 months

## Supplementary Table S12**: Model 2 statistics for the Mini Mental State Examination attention and calculation sub-scale**.

| Variable | β | [95% confidence interval] | | P value |
| --- | --- | --- | --- | --- |
| Age | -0.01 | -0.01 | -0.01 | <0.001 |
| Sex (female) | -0.08 | -0.12 | -0.03 | 0.001 |
| Education (secondary) | 0.21 | 0.15 | 0.26 | <0.001 |
| Education (third/higher) | 0.26 | 0.20 | 0.32 | <0.001 |
| Smoking status (smoker) | -0.11 | -0.19 | -0.03 | 0.006 |
| Body Mass Index (25-29.99) | -0.02 | -0.07 | 0.04 | 0.56 |
| Body Mass Index (30-39.99) | -0.03 | -0.09 | 0.04 | 0.42 |
| Body Mass Index (40+) | -0.17 | -0.39 | 0.06 | 0.14 |
| Depression (a major depressive  episode in the last 12 months) | -0.01683 | -0.14 | 0.11 | 0.80 |
| Total number of childhood infectious diseases | 0.03 | 0.01 | 0.06 | 0.01 |

Note.

N=2994

Model 2= the linear effect of total number of childhood infectious diseases, age, sex, education level, smoking status, Body Mass Index and depression on scores of the Mini Mental State Examination attention and calculation sub-scale.

Sex reference group = male

Education reference group = primary/none

Smoking status reference group = non-smoker

Body Mass Index reference group = 0-24.99

Depression reference group = not having had a major depressive episode in the last 12 months

## Supplementary Table S13: Model 2 statistics for the Mini Mental State Examination recall sub-scale.

| Variable | β | [95% confidence interval] | | P value |
| --- | --- | --- | --- | --- |
| Age | -0.02 | -0.03 | -0.02 | <0.001 |
| Sex (female) | 0.11 | 0.06 | 0.16 | <0.001 |
| Education (secondary) | 0.12 | 0.06 | 0.18 | <0.001 |
| Education (third/higher) | 0.26 | 0.20 | 0.32 | <0.001 |
| Smoking status (smoker) | -0.10 | -0.19 | -0.01 | 0.02 |
| Body Mass Index (25-29.99) | 0.01 | -0.04 | 0.07 | 0.69 |
| Body Mass Index (30-39.99) | -0.01 | -0.08 | 0.06 | 0.76 |
| Body Mass Index (40+) | -0.13 | -0.37 | 0.12 | 0.31 |
| Depression (a major depressive  episode in the last 12 months) | -0.06 | -0.20 | 0.08 | 0.39 |
| Total number of childhood infectious diseases | 0.04 | 0.02 | 0.07 | <0.001 |

Note.

N=2994

Model 2= the linear effect of total number of childhood infectious diseases, age, sex, education level, smoking status, Body Mass Index and depression on scores of the Mini Mental State Examination recall sub-scale.

Sex reference group = male

Education reference group = primary/none

Smoking status reference group = non-smoker

Body Mass Index reference group = 0-24.99

Depression reference group = not having had a major depressive episode in the last 12 months

## Supplementary Table S14: Model 2 statistics for the Mini Mental State Examination language sub-scale.

| Variable | β | [95% confidence interval] | | P value |
| --- | --- | --- | --- | --- |
| Age | -0.03 | -0.04 | -0.03 | <0.001 |
| Sex (female) | -0.01 | -0.07 | 0.045 | 0.63 |
| Education (secondary) | 0.27 | 0.20 | 0.35 | <0.001 |
| Education (third/higher) | 0.41 | 0.33 | 0.48 | <0.001 |
| Smoking status (smoker) | -0.12 | -0.22 | -0.01 | 0.03 |
| Body Mass Index (25-29.99) | 0.053 | -0.01 | 0.12 | 0.12 |
| Body Mass Index (30-39.99) | -0.002 | -0.08 | 0.08 | 0.96 |
| Body Mass Index (40+) | 0.08 | -0.21 | 0.36 | 0.60 |
| Depression (a major depressive  episode in the last 12 months) | -0.16 | -0.32 | 0.01 | 0.06 |
| Total number of childhood infectious diseases | 0.05 | 0.02 | 0.09 | <0.001 |

Note.

N=2994

Model 2 = the linear effect of total number of childhood infectious diseases, age, sex, education level, smoking status, Body Mass Index and depression on scores of the Mini Mental State Examination language sub-scale.

Sex reference group = male

Education reference group = primary/none

Smoking status reference group = non-smoker

Body Mass Index reference group = 0-24.99

Depression reference group = not having had a major depressive episode in the last 12 months

| Variable | β | [95% confidence interval] | | P value |
| --- | --- | --- | --- | --- |
| Age | -0.01 | -0.02 | -0.01 | <0.001 |
| Sex (female) | -0.003 | -0.08 | 0.08 | 0.85 |
| Education (secondary) | 0.12 | 0.02 | 0.22 | <0.001 |
| Education (third/higher) | 0.17 | 0.07 | 0.27 | <0.001 |
| Smoking status (smoker) | -0.11 | -0.25 | 0.03 | <0.001 |
| Body Mass Index (25-29.99) | 0.001 | -0.09 | 0.09 | 0.94 |
| Body Mass Index (30-39.99) | -0.02 | -0.13 | 0.09 | 0.36 |
| Body Mass Index (40+) | -0.10 | -0.53 | 0.27 | 0.25 |
| Depression (a major depressive  episode in the last 12 months) | -0.07 | -0.30 | 0.15 | 0.14 |
| Total number of childhood infectious diseases | 0.02 | -0.02 | 0.06 | 0.005 |

## Supplementary Table S15: Model 2 statistics for the dichotomized model (intact cognitive functioning vs. impaired cognitive functioning)

Note.

N=2994

Model 2= the linear effect of the number of childhood infectious diseases, age, sex, education level, smoking status, Body Mass Index and depression on intact vs. impaired cognitive functioning based on Mini Mental State Examination scores.

Sex reference group = male

Education reference group = primary/none

Smoking status reference group = non-smoker

Body Mass Index reference group = 0-24.99

Depression reference group = not having had a major depressive episode in the last 12 months

## Supplementary Table S16: Model 2 statistics without education

| Variable | β | [95% confidence interval] | | P value |
| --- | --- | --- | --- | --- |
| Age | -0.10 | -0.11 | -0.09 | <0.001 |
| Sex (female) | 0.08 | -0.07 | 0.23 | 0.29 |
| Smoking status (smoker) | -0.72 | -0.97 | -0.46 | <0.001 |
| Body Mass Index (25-29.99) | 0.02 | -0.15 | 0.18 | 0.86 |
| Body Mass Index (30-39.99) | -0.18 | -0.38 | 0.03 | 0.09 |
| Body Mass Index (40+) | -0.48 | -1.20 | 0.25 | 0.20 |
| Depression (a major depressive  episode in the last 12 months) | -0.41 | -0.81 | 0.003 | 0.05 |
| Total number of childhood infectious diseases | 0.23 | 0.15 | 0.31 | <0.001 |

Note.

N=2994

Model 2= the linear effect of the number of childhood infectious diseases, age, sex, smoking status, Body Mass Index and depression on Mini Mental State Examination scores.

Sex reference group = male

Smoking status reference group = non-smoker

Body Mass Index reference group = 0-24.99

Depression reference group = not having had a major depressive episode in the last 12 months

## Supplementary Table S17: Model 2 statistics for observed data with missing values

| Variable | β | [95% confidence interval] | | P value |
| --- | --- | --- | --- | --- |
| Age | -0.08 | -0.09 | -0.07 | <0.001 |
| Sex (female) | 0.10 | -0.04 | 0.25 | 0.16 |
| Education (secondary) | 0.70 | 0.52 | 0.87 | <0.001 |
| Education (third/higher) | 1.12 | 0.94 | 1.31 | <0.001 |
| Smoking status (smoker) | -0.46 | -0.71 | -0.21 | <0.001 |
| Body Mass Index (25-29.99) | 0.08 | -0.82 | 0.24 | 0.33 |
| Body Mass Index (30-39.99) | -0.04 | -0.24 | 0.15 | 0.66 |
| Body Mass Index (40+) | -0.19 | -0.90 | 0.52 | 0.59 |
| Depression (a major depressive  episode in the last 12 months) | -0.34 | -0.74 | 0.05 | 0.09 |
| Total number of childhood infectious diseases | 0.15 | 0.07 | 0.22 | <0.001 |

Note.

N=2803

Model 2= the linear effect of the number of childhood infectious diseases, age, sex, education level, smoking status, Body Mass Index and depression on Mini Mental State Examination scores.

Sex reference group = male

Education reference group = primary/none

Smoking status reference group = non-smoker

Body Mass Index reference group = 0-24.99

Depression reference group = not having had a major depressive episode in the last 12 months
